# Different visual manipulations have similar effects on quasi-static and dynamic balance responses of young and older people

**DOI:** 10.7717/peerj.11221

**Published:** 2021-05-11

**Authors:** Daniel Schmidt, Felipe P. Carpes, Thomas L. Milani, Andresa M.C. Germano

**Affiliations:** 1 Department of Human Locomotion, Institute of Human Movement Science & Health, Chemnitz University of Technology, Chemnitz, Germany; 2 Applied Neuromechanics Research Group, Federal University of Pampa, Uruguaiana, Brazil

**Keywords:** Visual information, Quasi-static balance, Dynamic balance, Age-related differences

## Abstract

**Background:**

Studies demonstrated that the older adults can be more susceptible to balance instability after acute visual manipulation. There are different manipulation approaches used to investigate the importance of visual inputs on balance, e.g., eyes closed and blackout glasses. However, there is evidence that eyes open versus eyes closed results in a different organization of human brain functional networks. It is, however, unclear how different visual manipulations affect balance, and whether such effects differ between young and elderly persons. Therefore, this study aimed to determine whether different visual manipulation approaches affect quasi-static and dynamic balance responses differently, and to investigate whether balance responses of young and older adults are affected differently by these various visual conditions.

**Methods:**

Thirty-six healthy participants (20 young and 16 older adults) performed balance tests (quasi-static and unexpected perturbations) under four visual conditions: Eyes Open, Eyes Closed, Blackout Glasses, and Dark Room. Center of pressure (CoP) and muscle activation (EMG) were quantified.

**Results:**

As expected, visual deprivation resulted in larger CoP excursions and higher muscle activations during balance tests for all participants. Surprisingly, the visual manipulation approach did not influence balance control in either group. Furthermore, quasi-static and dynamic balance control did not differ between young or older adults. The visual system plays an important role in balance control, however, similarly for both young and older adults. Different visual deprivation approaches did not influence balance results, meaning our results are comparable between participants of different ages. Further studies should investigate whether a critical illumination level may elicit different postural responses between young and older adults.

## Introduction

The interaction and integration of the afferent information allow the accomplishment of automated motor tasks (e.g., touch typing), as well as complex motor control (e.g., balance control after unexpected perturbation). In terms of quasi-static (When standing upright, there are still slight movements and oscillations of the upper body, for example. Hence, the term quasi-static balance is more appropriate than static balance, see also Chow, Lauk & Collins, 1999) and dynamic balance control, the center of pressure (COP) needs to be corrected to maintain or re-establish its position within the base of support (King, Judge & Wolfson, 1994). This control is more complex for dynamic balance, since two different postural strategies are necessary: anticipatory and compensatory responses (Santos, Kanekar & Aruin, 2010). While anticipatory adjustments are responsible for minimizing the forthcoming body perturbations, the compensatory responses should restore balance after a perturbation has already occurred (Santos, Kanekar & Aruin, 2010). In general, balance control responses originate from afferent information and the activation or inhibition of muscles involved in postural control. Therefore, the control of balance is known as a series of inputs and outputs of afferent and efferent information (acting-sensing-acting), which are generated to detect sway (e.g., COP or COM) and adjust motor actions continuously (e.g., muscle activities) (Peterka, 2002). Peterka (2002) affirmed that this control can be viewed functionally as a closed-loop feedback control system with the integration of different sources of sensory orientation inputs. In this way, it has already been established that balance control requires the complex integration of information from vestibular, visual, and somatosensory systems (Horak, Nashner & Diener, 1990). Although the information from each afferent system is extremely important for the control of balance, studies have not found a direct correlation between balance parameters and visual deprivation (Brocklehurst, Robertson & James-Groom, 1982) or a reduction of somatosensory sensory inputs (Anacker & FabioDi, 1992). Previous studies explained that, in case of a reduction of one or more sensory inputs, the remaining, intact systems are required to maintain balance (Horak, 2006). This means, redundant information from different receptors ensures that deficiencies in one or more input structure can be overcome by the remaining systems through reweighting or compensation (Toledo & Barela, 2014). This reweighing or compensation process is often recurred to maintain balance and can also be considered a safety mechanism when one system fails to provide the necessary information. However, based on our previous study (Germano, Schmidt & Milani, 2016), it seems that the modulation of reweighing depends on the type of task or level of difficulty. If the task does not challenge balance, e.g., quasi-static balance, no or small reweighting processes seem to be necessary in healthy individuals. On the other hand, for more challenging tasks, such as dynamic balance abilities, there seems to be an increase in recruitment of available afferent information to maintain balance (Germano, Schmidt & Milani, 2016).

Another point with regard to compensation processes is the availability and abundance of proper information. Age-induced structural and functional changes are directly related to receiving and integrating information. Declines in receptor concentrations (Shaffer & Harrison, 2007), number of synapses, axons, dendrites (Pannese, 2011), and signal transmission velocity (Lu et al., 2012) may elicit variability in balance among the elderly. As a consequence, age-related degeneration processes are known to induce balance instability (Fernández, Breinbauer & Delano, 2015).

With regard to the importance of visual information on balance, age-related degeneration processes have already been confirmed by innumerable studies (e.g., Pannese, 2011; Lu et al., 2012; Fernández, Breinbauer & Delano, 2015; Nagata et al., 2001; Hobeika, 1999). While some studies confirmed that balance responses are improved when combined with visual feedback (e.g., D’Anna et al., 2015), other studies examined the restricting effect visual information has on postural control (Ray et al., 2008; Schmid et al., 2007). However, there are some contradictions. For example, Hafström et al. (2002) suggested that the absence of visual information does not immediately alter the postural control as long as other sources of balance information are available. Another contradiction is related to the importance of visual inputs at different ages. A recent study found that elderly adults (compared to young adults) are particularly dependent on vision to maintain postural stability (Osoba et al., 2019). In contrast, there are studies showing no differences of postural outcomes comparing young versus older subjects when vision was deprived (e.g., Hytönen et al., 2009).

In general, although in healthy subjects vision cannot be totally disconnected, manipulating visual inputs is essential to understanding balance behavior during changes in visual information. In this way, researchers have tried to reduce, manipulate, or eliminate visual inputs in healthy subjects using different procedures like eyes closed, the use of bandages (Candido et al., 2012), gauze pads (Vig, Showfety & Phillips, 1980), or the use of blackout glasses (Boroojerdi et al., 2000; Merabet et al., 2008). There are also studies which do not specify how the visual deprivation was accomplished (e.g., Iosa et al., 2012). However, typical everyday situations regarding an acute deprivation of visual information during a balance task often occur with eyes open in darkness. For example, Berg et al. (1997) showed that approximately 20% of falls occur at evening and night, and of those, most occur between 9 pm and 7 am. To date, however, only few studies have compared balance aspects when performed with different visual manipulations (Hafström et al., 2002). Furthermore, standing with eyes closed or eyes open in darkness should be equivalent with respect to the amount of visual information available (Hafström et al., 2002). However, there are two different states of mental activity when the eyes are open or closed. An “interoceptive” state is established with closed eyes, characterized by imagination and sensory activity, while the activation of attentional and ocular motor structures with open eyes induces an “exteroceptive” state (Marx et al., 2003). Taking this into consideration, it can be suggested that the integration process of afferent inputs during balance may differ when the task is performed with eyes open (in darkness) or closed, possibly causing differences in balance control. If this is true, the various experimental approaches to reduce or manipulate visual input (e.g., eyes closed vs. blackout glasses but eyes open) may result in different motor outcomes as a consequence of merely opening the eyes. In case there are differences between the processing of visual information following different visual conditions during balance, it might be difficult to interpret and compare the results obtained. Additionally, possible age-related differences regarding the availability of afferent information or “efficiency” of integration could also induce changes in balance control (COP and muscle activities) under various visual manipulations, and/or with different levels for balance tasks (Prioli, Freitas & Barela, 2005; Prioli et al., 2006).

Therefore, we aimed to determine whether (1) different approaches of visual manipulation affect quasi-static and dynamic balance responses differently, and (2) whether balance responses of young and older adults are affected differently by various visual manipulations. We hypothesized greater instability (significant increases in both COP excursions and electromyographic (EMG) signals) for the visual manipulation conditions compared to eyes open for both age groups. Additionally, changes within the three analyzed visual manipulation conditions (Eyes Closed, Blackout Glasses, Dark Room) were hypothesized. Finally, we expected greater instability for older compared to younger adults for all conditions.

## Methods

### Participants

Sample size was determined based on previous studies using G*Power software (version 3.1.2, Kiel University, Germany). Thirty-six participants (20 female, 16 male) were organized into two age groups: 20 young (11 female, 9 male; mean ± SD: 22.9 ± 2.5 yrs, range: 18–35 yrs, 70.9 ± 11.8 kg, 1.8 ± 0.08 m) and 16 older adults (9 female, 7 male; mean ± SD: 69.3 ± 4.2 yrs, range: 60–80 yrs, 73.8 ± 10.4 kg, 1.7 ± 0.1 m). A questionnaire was used to evaluate the type and frequency of physical activity performed by the subjects. Most participants (31 participants) exercised regularly at the time of the experiments, details can be found in Table 1. No participants had lower-extremity injuries for at least the previous six months, neurological disorders, mild visual impairment (Stage 1 according to World Health Organization), claustrophobia, or were taking medication that could affect balance. They were informed about the purpose of this study and provided written consent. All procedures were conducted according to the Declaration of Helsinki and were approved by the ethics committee of the Faculty of Behavioral and Social Sciences at the Chemnitz University of Technology (V-191-17-TM-Blind-05042017).

**Table 1:** Profile of the sportive activities of the participants who took part in this study.

Sports activities per week (*n*)						1–3	≥4
	Young (*n* = 20, in %)					50	50
	Older (*n* = 16, in %)					65	35
**Types of sports activities (in %)**							
Weight Training	Water Sports	Ball Sports	Individual Sports	Senior Sports	Dance	Running	Gymnastics
26	18	15	15	9	9	4	4

### Apparatus

**Figure 1:**
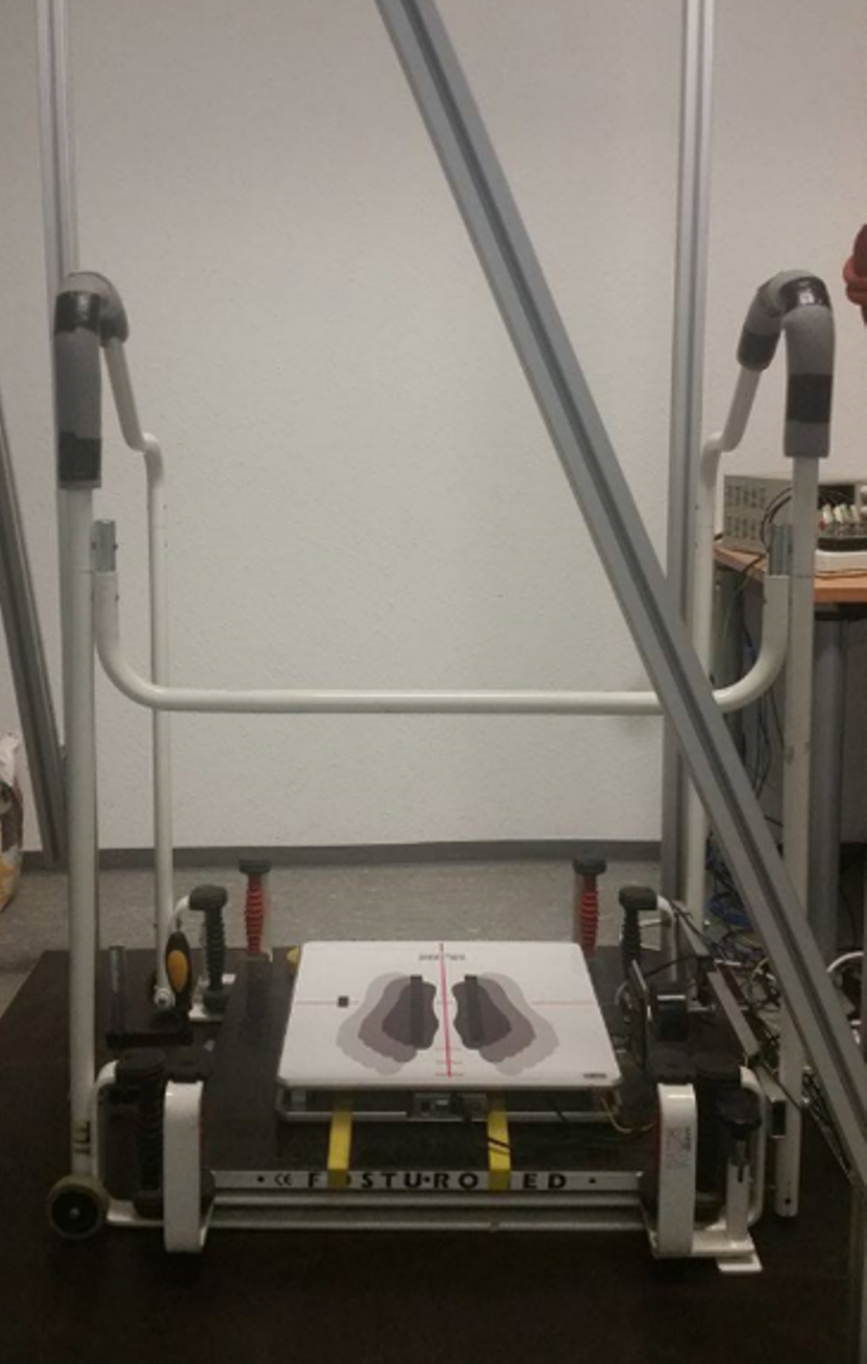
Photograph of the setup of the balance tests. Instrumentation to conduct quasi-static (bottom platform fixed) and dynamic (bottom platform attached to the electro-magnet in order to allow sudden perturbations after its release) balance tests. The electro-magnet is located to the right of the force platform.

A force plate (IMM Holding GmbH, Germany; sampling rate 1 kHz) was used to quantify quasi-static and dynamic balance ability. The force plate was attached directly on top of the bottom platform of the commercially-available Posturomed device (Haider Bioswing GmbH, Germany, Fig. 1). The Posturomed device consists of a vertically suspended horizontally mobile bottom-platform, for which the level of mobility can be adjusted by locking or unlocking eight springs. This device enables the quantification of dynamic postural control (Germano, Schmidt & Milani, 2016; Kiss, 2011; Schmidt, Germano & Milani, 2015) by calculating center of pressure parameters (see 2.5 Data analyses) based on vertical force data. To perform quasi-static balance tests, the bottom-platform of the Posturomed was fully fixed, avoiding any movement. For the dynamic balance measurements, unexpected horizontal perturbations were induced using an electro-magnet attached to the bottom platform. The easiest level of difficulty of the Posturomed device was used in all dynamic measurements, blocking the four black springs and only releasing the four red springs. After pressing a manual trigger, the bottom platform was released 20 mm out of the neutral position thus initiating an unexpected perturbation (Germano, Schmidt & Milani, 2016; Schmidt, Germano & Milani, 2015). Successively, the bottom platform swung horizontally until it reached the neutral position. The trigger button was pressed once for each trial, which lasted a total of 500 ms (analyzed intervals are described in 2.5 Data analyses). Additionally, a single axis accelerometer (ADXL78, Analog Devices Inc., USA; sampling rate 1 kHz) was fixed to the bottom platform to detect the reversal points of the oscillations after triggering. All participants were secured using a safety belt, and the handrail of the Posturomed was covered with insulation material to avoid injuries. The whole balance setup was located in a room that could be completely darkened. The devices to collect data were located in an adjacent room. Communication between both rooms was performed using an infrared camera, a microphone, and a loudspeaker.

### Electromyography (EMG)

Muscle activities of the tibialis anterior (TA) and the gastrocnemius medialis (GM) from the preferred leg (determined by the preference to kick a ball (Harris, 1957)) were measured using wireless bipolar surface electrodes (Trigno™ Wireless, Delsys Inc., USA; DC-500 Hz, 160 dB/Dec., sampling rate of 2 kHz). EMG electrodes were positioned according to the recommendations of SENIAM (Hermens et al., 1999). Skin preparation included shaving, abrasion by sandpaper, and cleaning with alcohol pads.

### Testing procedure

Prior to the tests, all participants were given an acclimatization period of 10 min and performed at least three training trials of the dynamic balance tasks to become familiar with the apparatus. Before data collection and for each condition, room temperature was monitored (by using a digital C28 type K thermocouple, Comark Instruments, U.K.) to keep the room temperature at 23 ±  2^∘^C (according to EN ISO/IEC 17025) to avoid temperature changes. The same thermometer was used to measure the foot sole skin temperature at the heel of the preferred foot to monitor possible relevant changes. Quasi-static and dynamic balance abilities were tested in a randomized order, and all balance tests were performed in double leg stance. The protocol consisted of three trials for each balance test (quasi-static balance and dynamic balance in anterior-posterior and medio-lateral directions) and for each randomized condition: Eyes Open (EO), Eyes Closed (EC), Blackout Glasses (BG), and Dark Room (DR). The experimental room used normal illumination during EO, BG, and EC conditions. There was no visible light source during DR conditions. Participants kept their eyes open for BG and DR conditions. For the quasi-static balance tests, trials of 25s were performed and participants were instructed to keep their knees straightened but not locked, and to keep their upper limbs hanging down. They were also asked to evenly distribute their body weight on both feet (shoulder width apart). For the dynamic balance test, trials of unexpected perturbations were performed in anterior-posterior and medio-lateral directions, and the participants were also asked to maintain the above-mentioned posture. The preferred leg was always positioned toward the electro-magnet which induced the perturbation. This guaranteed that the preferred leg had the same importance with respect to the dynamic balance task. The subjects indicated verbally when they were prepared to perform the next trial, hence, resting periods were allowed to the subjects. Exclusion criteria for the trials were: participants touched the handrail; lost their concentration (e.g., talking, moving); or changed the foot position.

### Data analyses

A routine written in LabView 8.0 (National Instruments Corp., USA) recorded and synchronized the data. Dynamic balance data were synchronized using a manual trigger (onset of unexpected perturbation). All data were processed using a routine written with R (The R Foundation for Statistical Computing, Austria). Force plate data were low-pass filtered (10 Hz, 4th order Butterworth filter) and used to quantify balance ability through center of pressure (COP) total excursion and its medio-lateral (ML) as well as anterior-posterior (AP) lengths based on the Pythagorean Theorem. Such parameters were frequently used in previous studies (e.g., Prasad et al., 2011; Schmidt, Germano & Milani, 2015; Era & Heikkinen, 1985; Sundermier et al., 1996). Furthermore, force plate data were normalized by the weight of the participants since the mass of the participants influenced the path length of the signal. For quasi-static balance, the first 5 s of the trial were discarded. For dynamic balance tasks (each trial lasted 5 s), data were analyzed from three time intervals to assess anticipatory and compensatory phases in relation to the trigger (T_0_): −200 ms to T_0_ (interval 0); T_0_ to 70 ms post-trigger (interval 1); and 71 ms to 260 ms post-trigger (interval 2) as described elsewhere (Schmidt, Germano & Milani, 2015). The average velocity of the moveable platform was 0.1413 ±  0.0006 m/s and 0.1528 ±  0.0005 m/s for intervals 1 and 2, respectively. EMG data were offset-corrected, rectified, and band-pass filtered (20–500 Hz; Butterworth 4th order). Muscle activation was quantified by root mean square (RMS) values.

### Statistical analyses

The three trials from each participant were averaged for each condition and each balance ability. Normal distribution was checked using the Shapiro–Wilk test (*α* = 0.05). Differences between the visual conditions for the normally distributed data (COP total excursion data of young group during quasi-static balance) were examined using ANOVA for repeated measures with Bonferroni post-hoc. Friedman tests following a Wilcoxon test were carried out for the non-normally distributed data (all other COP data and all EMG data). Since non-normally distributed data were present, group differences were identified using the Kruskal-Wallis test following Mann–Whitney-*U* tests. A Bonferroni correction was used to adjust the level of significance (*α* = 0.05/4 = 0.0125) to account for the four conditions. Effect sizes (*r*) were also calculated.

## Results

### Quasi-static balance

**Figure 2:**
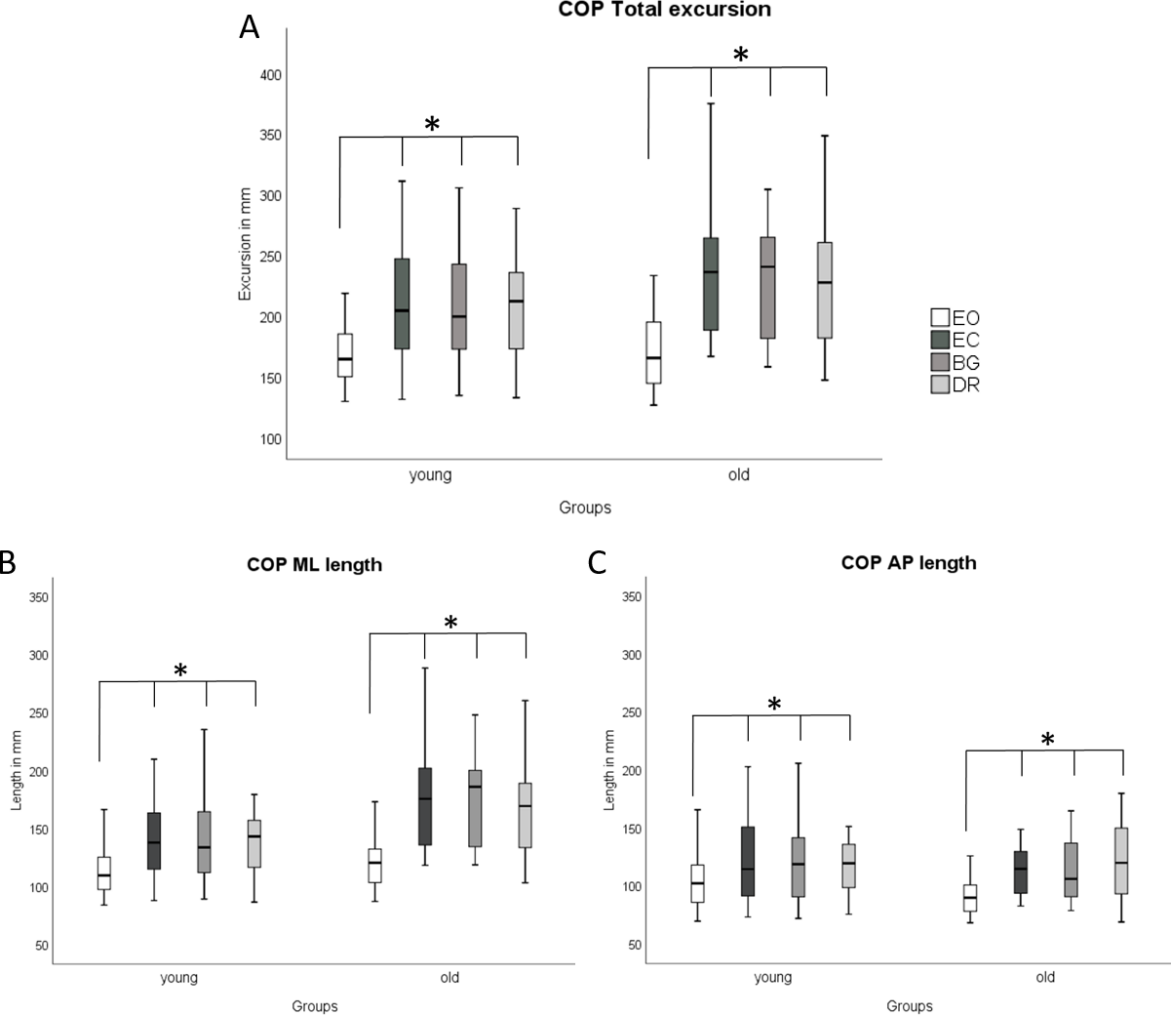
Boxplots of COP results during quasi-static balance. COP results ((A) COP Total Excursions, (B) COP ML Length, (C) COP AP Length) are shown during quasi-static balance for each condition (Eyes Open, Eyes Closed, Blackout Glasses, Dark Room: EO, EC, BG, DR) and each group (young adults and older adults). * Significant differences (∗*p* ≤ 0.0125) were present when comparing EO to all visual manipulation conditions. * Significant differences (∗*p* ≤ 0.0125) were found for the following comparisons: Within young adults: COP Total: EO vs. EC (*p* < 0.001; *r* =  − 0.61); EO vs. BG (*p* < 0.001; *r* =  − 0.58); EO vs. DR (*p* < 0.001; *r* =  − 0.61), ML length; EO vs. EC (*p* < 0.001; *r* =  − 0.62); EO vs. BG (*p* < 0.001; *r* =  − 0.62); EO vs. DR (*p* < 0.001; *r* =  − 0.62)/ AP length: EO vs. EC (*p* < 0.001; *r* =  − 0.56); EO vs. BG (*p* < 0.001; *r* =  − 0.48); EO vs. DR (*p* < 0.001; *r* =  − 0.47). Within older adults: COP Total: EO vs. EC (*p* < 0.001; *r* =  − 0.62); EO vs. BG (*p* < 0.001; *r* =  − 0.62); EO vs. DR (*p* < 0.001; *r* =  − 0.61)/ ML length: EO vs. EC (*p* < 0.001; *r* =  − 0.62); EO vs. BG (*p* < 0.001; *r* =  − 0.62); EO vs. DR (*p* < 0.001; *r* =  − 0.61)/AP length: EO vs. EC (*p* < 0.001; *r* =  − 0.62); EO vs. BG (*p* < 0.001; *r* =  − 0.62); EO vs. DR (*p* < 0.001; *r* =  − 0.60).

**Figure 3:**
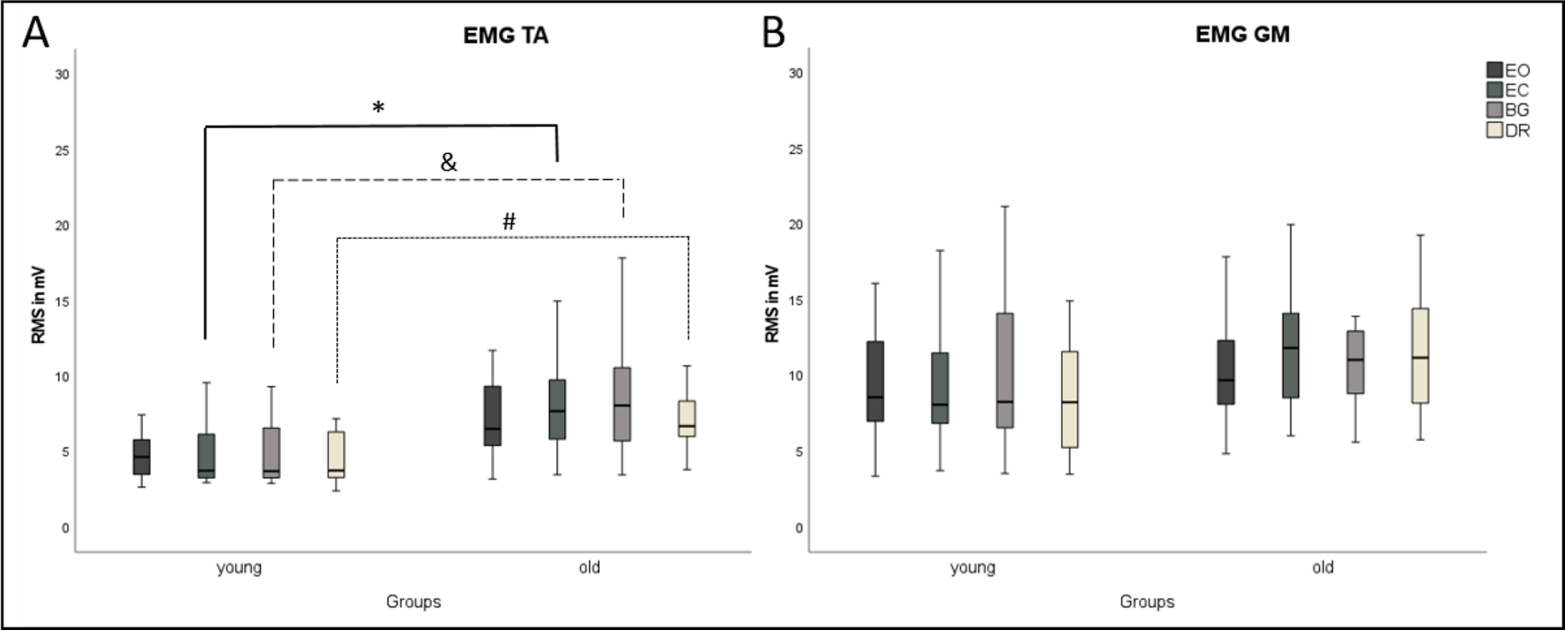
Boxplots of EMG results during quasi-static balance. EMG results ((A) TA, Tibialis anterior; (B) GM, Gastrocnemius medialis) are shown for each condition (Eyes Open, Eyes Closed, Blackout Glasses, Dark Room: EO, EC, BG, DR) and each group (young adults and older adults). ^∗,&,#^ Significant differences (*p* ≤ 0.0125). TA, M. tibialis anterior; GM, M. gastrocnemius medialis.

COP parameters and EMG did not differ between the three visual deprivation conditions for either age group (Figs. 2 and 3). As expected, COP parameters were lower during EO compared to all visual manipulation conditions, regardless of participant age. For TA, younger subjects showed significantly lower RMS values (*p* ≤ 0.0125) than older adults for EC, BG, and DR, but not for EO (Fig. 3). There were no age-related RMS differences for GM (Fig. 3).

### Dynamic balance

#### Anterior-posterior perturbation

There were no differences between the three visually deprived conditions for either group (Table 2). EO differed from the deprived conditions. Anticipatory (interval 0) COP excursions were larger for all visually deprived conditions compared to EO, and AP length was longer for DR compared to EO (Table 2). No difference was found for interval 1. Compensatory responses (interval 2) showed shorter ML length in EO compared to BG. For older subjects, intervals 0 and 2 showed differences between EO versus EC or BG. Young and older subjects did not differ for any parameters, conditions, or intervals.

Muscle activation in EO did not differ from the visually deprived trials in young participants (Table 2). Among older participants, differences in anticipatory responses (intervals 0 and 1) were mainly found when comparing EO vs. BG or EC. Differences between the visually deprived conditions were only found between BG vs. DR for TA in interval 1. For compensatory responses (interval 2), no differences were found. Similarly to COP parameters, there were no differences between age groups for RMS GM. Muscle activation of TA was significantly larger for the older group compared to the young group in five out of 12 comparisons (Table 2).

**Table 2:** Mean ± standard-deviation for COP (in mm) and EMG (in mV) parameters during dynamic balance in the anterior-posterior perturbation direction. Parameters are shown for each condition (Eyes Open, Eyes Closed, Blackout Glasses, Dark Room: EO, EC, BG, DR) during dynamic balance in the anterior-posterior perturbation direction for intervals 0, 1, and 2 comparing both groups (young adults and older adults). TA: M. tibialis anterior; GM: M. gastrocnemius medialis. Significant differences (*p* ≤ 0.0125) and effect sizes are also presented for the comparison between the conditions.

AP perturbation direction						
	Groups	EO	EC	BG	DR	*p*-value and effect size for sig. Differences
COP Total	young	2.35 ± 0.79	2.97 ± 0.83	3.22 ± 1.37	3.25 ± 2.16	EO vs. EC (*p* < 0.001; *r* = − 0.45), EO vs. BG (*p* < 0.001; *r* = − 0.45), EO vs. DR (*p* = 0.01; *r* = − 0.44)
Int 0	old	2.33 ± 0.55	3.23 ± 1.07	3.16 ± 0.99	2.95 ± 1.32	EO vs. EC (*p* = 0.01; *r* = − 0.70), EO vs. BG (*p* = 0.01; *r* = − 0.72)
COP Total	young	8.35 ± 1.32	8.66 ± 1.45	8.21 ± 1.38	8.48 ± 1.53	
Int 1	old	8.54 ± 1.29	8.86 ± 1.25	8.64 ± 1.15	8.74 ± 1.36	
COP Total	young	38.09 ± 13.79	40.51 ± 15.41	38.26 ± 13.45	40.97 ± 11.24	
Int 2	old	36.44 ± 8.57	42.42 ± 10.03	42.04 ± 9.9	40.39 ± 10.18	EO vs. EC (*p* < 0.001; *r* = − 0.76), EO vs. BG (*p* < 0.001; *r* = − 0.74)
ML length	young	1.35 ± 0.55	1.58 ± 0.52	1.61 ± 0.60	1.58 ± 0.67	
Int 0	old	1.18 ± 0.39	1.58 ± 0.64	1.54 ± 0.56	1.55 ± 0.80	EO vs. EC (*p* = 0.01; *r* = − 0.50), EO vs. BG (*p* < 0.001; *r* = − 0.52)); GM (EO vs. BG (*p* < 0.001; *r* = − 0.59))
ML length	young	2.89 ± 0.68	2.96 ± 0.67	2.87 ± 0.54	2.76 ± 0.66	
Int 1	old	2.94 ± 0.48	3.02 ± 0.50	3.07 ± 0.47	2.97 ± 0.35	
ML length	young	13.74 ± 7.01	13.71 ± 7.21	13.34 ± 8.57	14.22 ± 5.55	EO vs. BG (*p* < 0.001; *r* = − 0.45)
Int 2	old	12.50 ± 3.41	14.16 ± 5.10	13.08 ± 3.55	12.91 ± 4.34	
AP length	young	1.59 ± 0.63	2.14 ± 0.79	2.41 ± 1.33	2.42 ± 2.10	EO vs. DR (*p* = 0.01; *r* = − 0.43)
Int 0	old	1.73 ± 0.52	2.42 ± 0.91	2.41 ± 0.96	2.12 ± 1.19	EO vs. EC (*p* = 0.01; *r* = − 0.49)
AP length	young	7.23 ± 1.40	7.52 ± 1.51	7.10 ± 1.51	7.44 ± 1.66	
Int 1	old	7.39 ± 1.35	7.68 ± 1.40	7.42 ± 1.24	7.58 ± 1.45	
AP length	young	32.75 ± 12.60	35.49 ± 13.67	33.16 ± 10.63	35.30 ± 11.60	
Int 2	old	31.78 ± 8.68	37.33 ± 9.16	37.51 ± 9.76	35.95 ± 9.44	EO vs. EC (*p* < 0.00; *r* = − 0.63), EO vs. BG (*p* < 0.00; *r* = − 0.60), EO vs. DR (*p* = 0.01; *r* = − 0.46)
RMS TA	young	3.66 ± 1.16^*^	4.26 ± 1.99^*^	3.97 ± 1.64^*^	4.04 ± 2.52	
Int 0	old	6.29 ± 4.31^*^	8.94 ± 7.78^*^	6.41 ± 2.90^*^	5.77 ± 2.84	EO vs. BG (*p* < 0.001; *r* = − 0.51)
RMS TA	young	3.63 ± 1.13	4.51 ± 3.05	3.92 ± 1.62^*^	3.72 ± 1.33	
Int 1	old	5.50 ± 3.29	8.87 ± 8.86	9.70 ± 9.89^*^	5.63 ± 2.46	EO vs. BG (*p* = 0.01; *r* = − 0.46), BG vs. DR (*p* = 0.01; *r* = − 0.48)
RMS TA	young	68.33 ± 38.71	74.17 ± 34.97^*^	67.56 ± 28.75	72.55 ± 32.75	
Int 2	old	95.99 ± 40.71	125.43 ± 56.14^*^	96.48 ± 37.47	116.97 ± 55.81	
RMS GM	young	8.82 ± 4.78	13.06 ± 11.44	13.13 ± 8.80	11.05 ± 6.39	
Int 0	old	8.57 ± 2.99	9.97 ± 3.88	10.97 ± 4.68	10.37 ± 5.31	EO vs. BG (*p* = 0.01; *r* = − 0.46)
RMS GM	young	8.07 ± 4.80	11.34 ± 8.99	11.50 ± 7.92	8.75 ± 5.09	
Int 1	old	8.33 ± 3.74	10.25 ± 3.26	11.33 ± 4.20	10.76 ± 5.40	EO vs. EC (*p* = 0.01; *r* = − 0.47)
RMS GM	young	11.16 ± 6.28	13.48 ± 8.47	13.43 ± 7.76	13.76 ± 9.56	
Int 2	old	13.97 ± 6.74	15.42 ± 6.61	16.90 ± 7.44	15.54 ± 7.79	

**Notes.**

* Significant differences between the groups are indicated with “*” (^∗^*p* ≤ 0.0125).

#### Medio-lateral perturbation

Medio-lateral perturbation elicited differences in COP parameters between EO and the visually deprived conditions, but no differences were found between visually deprived conditions (Table 3). These differences occurred in interval 0 for the young participants, showing larger COP excursions for the deprived conditions compared to EO (ML length: EO vs. BG; AP length: EO vs. DR) (Table 3). Among older participants, differences were found in interval 0 (AP length: smaller excursion for EO compared to DR) and interval 2 (ML length: smaller excursions for EO compared to BG). COP parameters showed no difference between young and older participants.

**Table 3:** Mean ± standard-deviation for COP (in mm) and EMG (in mV) parameters during dynamic balance in the medio-lateral perturbation direction. Parameters are shown for each condition (Eyes Open, Eyes Closed, Blackout Glasses, Dark Room: EO, EC, BG, DR) during dynamic balance in the medio-lateral perturbation direction for intervals 0, 1, and 2 comparing both groups (young adults and older adults). TA, M. tibialis anterior; GM, M. gastrocnemius medialis. Significant differences (*p* ≤ 0.0125) and effect sizes are also presented for the comparison between the conditions.

ML perturbation direction						
	Groups	EO	EC	BG	DR	*p*-value and effect size for sig. Differences
COP Total	young	2.53 ± 0.96	2.98 ± 0.94	3.29 ± 1.42	3.06 ± 0.94	
Int 0	old	2.61 ± 1.04	3.15 ± 0.94	3.54 ± 1.8	3.74 ± 1.65	
COP Total	young	7.2 ± 1.23	7.29 ± 1.27	7.18 ± 1.12	7.03 ± 1.13	
Int 1	old	6.88 ± 0.62	7.09 ± 0.85	7.11 ± 1.12	6.92 ± 0.61	
COP Total	young	51.3 ± 12.74	53.61 ± 13.55	55.38 ± 16.49	57.09 ± 13.51	
Int 2	old	42.86 ± 11.99	45.63 ± 12.37	47.45 ± 12.45	48.51 ± 13.92	
ML length	young	1.45 ± 0.81	1.72 ± 0.75	1.91 ± 1.07	1.63 ± 0.78	EO vs. BG (*p* = 0.01; *r* = − 0.41)
Int 0	old	1.41 ± 0.70	1.56 ± 0.67	1.43 ± 0.60	1.89 ± 1.13	
ML length	young	5.70 ± 1.17	5.73 ± 1.13	5.62 ± 1.14	5.55 ± 1.17	
Int 1	old	5.29 ± 0.64	5.42 ± 0.76	5.48 ± 1.13	5.37 ± 0.64	
ML length	young	44.68 ± 11.17	47.33 ± 12.61	48.25 ± 14.54	50.33 ± 12.59	
Int 2	old	36.74 ± 10.86	39.41 ± 10.93	42.03 ± 11.98	42.48 ± 11.39	EO vs. BG (*p* < 0.001; *r* = − 0.52)
AP length	young	1.70 ± 0.63	1.98 ± 0.66	2.18 ± 0.87	2.20 ± 0.67	EO vs. DR (*p* = 0.01; *r* = − 0.42)
Int 0	old	1.90 ± 0.75	2.38 ± 0.76	2.88 ± 1.80	2.74 ± 1.45	EO vs. DR (*p* = 0.01; *r* = − 0.49)
AP length	young	3.17 ± 0.83	3.28 ± 0.85	3.25 ± 0.66	3.14 ± 0.54	
Int 1	old	3.26 ± 0.54	3.39 ± 0.73	3.31 ± 0.77	3.23 ± 0.51	
AP length	young	16.85 ± 7.09	16.37 ± 5.54	17.74 ± 8.19	18.06 ± 5.08	
Int 2	old	16.57 ± 6.22	17.57 ± 6.50	16.45 ± 5.28	17.65 ± 9.91	
RMS TA	young	3.81 ± 1.31^*^	3.98 ± 1.28	3.92 ± 1.65^*^	3.64 ± 0.97^*^	
Int 0	old	8.85 ± 8.98^*^	13.45 ± 16.79	7.58 ± 5.12^*^	7.52 ± 6.29^*^	
RMS TA	young	3.87 ± 1.33	4.66 ± 2.99^*^	3.99 ± 1.67^*^	3.66 ± 1.12^*^	
Int 1	old	8.03 ± 7.35	12.64 ± 14.68^*^	7.40 ± 5.19^*^	7.63 ± 5.46^*^	
RMS TA	young	27.10 ± 15.48^*^	30.27 ± 15.13^*^	37.34 ± 21.83^*^	35.96 ± 20.87^*^	EO vs. DR (*p* = 0.01; *r* = − 0.41)
Int 2	old	62.51 ± 33.81^*^	69.33 ± 33.58^*^	76.86 ± 31.90^*^	78.65 ± 32.83^*^	EO vs. DR (*p* = 0.01; *r* = − 0.42)
RMS GM	young	7.62 ± 5.03	10.77 ± 7.17	10.77 ± 8.82	10.78 ± 7.12	
Int 0	old	8.21 ± 3.91	9.33 ± 3.17	9.41 ± 4.86	9.63 ± 5.90	
RMS GM	young	6.43 ± 3.87	8.79 ± 5.03	10.75 ± 7.90	11.15 ± 7.78	EO vs. BG (*p* = 0.01; *r* = − 0.48), EO vs. DR (*p* = 0.01; *r* = − 0.44)
Int 1	old	8.21 ± 4.08	10.00 ± 3.75	8.20 ± 3.56	9.89 ± 4.13	
RMS GM	young	28.23 ± 17.99	33.98 ± 22.08	35.26 ± 25.93	40.12 ± 27.33	
Int 2	old	29.32 ± 11.12	37.30 ± 15.01	32.94 ± 11.72	35.89 ± 17.26	EO vs. EC (*p* = 0.01; *r* = − 0.47)

**Notes.**

* Significant differences between the groups are indicated with “*” (^∗^*p* ≤ 0.0125).

Muscle activation did not differ within the visually deprived conditions, but differences were found comparing these conditions to EO: Interval 0 exhibited no differences for both muscles and both age groups. For interval 1, EO consistently resulted in lower GM activation compared to BG and DR for young participants only. In interval 2, greater TA activation was found for the visually deprived conditions (EO compared to DR) for both age groups. In older participants, differences were also found for interval 2, showing lower GM activation for EO compared to EC. Similarly to the AP perturbation direction, significant differences between both age groups were only found for TA (10 out of 12 comparisons) and not for GM.

## Discussion

Effects of different visual deprivation approaches (Eyes Closed, Blackout Glasses, Dark Room) on quasi-static and dynamic balance in young and older adults were investigated. We hypothesized different responses within the three visual manipulation conditions. Moreover, we presumed greater instability for the visual deprivation conditions compared to eyes open. Finally, we expected greater instability for older compared to younger adults.

### Balance control during visual deprivation and eyes open

Our results generally showed no differences in quasi-static and dynamic balance when comparing the different strategies of visual deprivation (EC vs. BG vs. DR). We did not expect this finding for two reasons. First, changes in affferent information were shown to result in changes in balance responses (Magnusson, 2016), in our case this is related to visual inputs. Second, comparisons between EO in complete darkness (in our study DR) and EC have shown different brain activations (Marx et al., 2003; Xu et al., 2014). The authors of these studies investigated human brain functional networks using resting-state functional magnetic resonance imaging and neural network analysis for comparing eyes open (in complete darkness) and eyes closed in young participants. When the eyes are open but in complete darkness, attentional and oculomotor systems are activated, while sensory systems are enabled when the eyes are closed (Marx et al., 2003; Xu et al., 2014). This led us to presume that increased activation of other sensory systems with closed eyes could increase postural control compared to having eyes open in darkness. Hence, we also expected differences between BG and EC, since eyes were open in the former condition. This was not confirmed by our data. We need to consider that the two studies mentioned above were not performed during balance tasks, but that participants performed the measurements in a supine position, which may have influenced the outcomes.

The findings of our study are in line with a previous examination in which EO in darkness was compared to EC not only during quiet stance, but also during more demanding balance situations (vibratory stimulations at the calf muscles) in three of four analyzed periods in young subjects (Hafström et al., 2002). In contrast to our findings, however, this study (Hafström et al., 2002) showed differences when comparing eyes open in darkness and eyes closed during the first period of vibratory stimulation. The authors explained this by a longer latency in the balance control system to verify whether or not visual information is available with eyes open in darkness. Since our results showed that balance performance did not change within the three visually deprived conditions for both age groups and all parameters, we presume that different visual manipulation protocols are comparable and, therefore, each analyzed visual manipulation can be used in future studies.

When comparing eyes open with the other visual conditions (EC, BG, and DR) to examine balance control, as expected, eyes open resulted in better postural control compared to the three visually deprived conditions during quasi-static and dynamic balance (especially in intervals 0 and 2) or postural-motoric situations in general. This is in agreement with several other studies (e.g., Iosa et al., 2012; Ernst & Bülthoff, 2004). However, there are also studies showing no differences of quasi-static postural control when comparing EO versus EC in young (Hytönen et al., 2009; Hafström et al., 2002) and older subjects (61–75 yrs) (Hytönen et al., 2009).

The majority of our COP findings support our hypothesis, confirming the importance of visual information during quiet stance for both age groups. This can be explained by the so-called reweighing process in postural control. In order to control balance, sensory signals may stem from visual, vestibular, proprioceptive, and plantar skin sensitivity inputs, also called non-redundant sensory signals (Ernst & Bülthoff, 2004). According to Ernst & Bülthoff (2004), those signals need to be combined resulting in redundant signals during postural control. For example, visual information needs to be combined with certain proprioceptive information to enable standardized body coordinates. Note that such sensory combinations have already been shown to occur in early stages of signal processing at the spinal cord level (Lowrey & Bent, 2009; Germano, Schlee & Milani, 2016). In case of lost or diminished information from one or more afferent receptors, as in the visually deprived situations in the present study, the central nervous system alters the processing of afferent information to maintain balance and the remaining, intact systems may compensate this absence using reweighing processes to maintain balance (Brocklehurst, Robertson & James-Groom, 1982). Hence, the increases in COP excursions reflect balance instability, but resulted in a successful compensation when the vision was deprived, as our participants were able to maintain balance.

Muscle activation in quasi-static balance, however, did not differ between EO and the visually deprived conditions. Therefore, this does not agree with our COP data. In order to examine this further, we conducted a correlation analyses between our EMG and COP data which confirms that these parameters do not seem to be related to each other. There are some possible explanations for this. The first explanation is that certain changes in COP data do not necessarily reflect changes in EMG data (Billot et al., 2013), especially due to possible different latencies between COP and muscle activities. In this case, there is simply no obvious correlation. However, it should also be mentioned that our subjects may have used the so-called hip strategy to control balance, as described by Winter et al. (1996), when measuring balance control in the frontal plane. Hence, the COP changes may also result in the activation of other muscles not evaluated here (e.g., intrinsic foot muscles) (Kelly et al., 2012). Unfortunately, muscle activity associated with the hip strategy or intrinsic foot muscles was not measured, which is clearly a limitation of our study. However, there is also another study (Chang et al., 2005) that did not find a correlation between the rate of force production and COP parameters during compensatory stepping, which is a dynamical task. Interestingly, the same study found a correlation when quasi-static balance conditions were examined. Finally, it should also be mentioned that it is not as clear as it might seem regarding which postural strategy (ankle vs. hip strategy) might have been selected from our participant for these more simple quasi-static tests: Although it is a general understanding that the more demanding tasks activate hip muscles (hip strategy) and low postural demands activate the ankle muscles (Horak, Nashner & Diener, 1990; Horak & Nashner, 1986), it is hard to “draw a line”, as those strategies may also work synergistically. All in all, it remains unclear why we did not find correlations between our EMG and COP parameters. To summarize our findings, although quasi-static balance is considered a low postural demand, the visual manipulation was sufficient to induce higher COP excursions. However, no significant increases in muscle activation (Tib and GM) were detected.

For dynamic balance, larger muscle activation was found during visual deprivation compared to EO, which may suggest an additional neuromuscular demand to compensate perturbations. Prior to any changes in COP, muscular contractions must be present to shift the COP. Hence, it would be plausible that muscle activation starts in interval 1 and induces COP shifts in interval 2 due to the latency (Germano, Schmidt & Milani, 2016). During anterior-posterior perturbations, only older participants exhibited differences in EMG when comparing EO versus EC, BG, and DR. During bipedal stance, the distance between the most anterior and posterior parts of the foot sole (sagittal plane) may be narrower than the distance between the most lateral aspects (frontal plane). This would cause a greater challenge for anterior-posterior perturbations compared to ML, possibly requiring increased muscle activity in our older group, especially during the deprived conditions. In contrast to anterior-posterior perturbations, during medio-lateral perturbations also young participants showed differences between the visually deprived conditions vs. EO. It remains unclear why the young group showed greater muscle activation during medio-lateral perturbations for the visually deprived conditions compared to EO. It could be important to analyze these effects in light of muscle strength and/or maximum upright leaning positions as this may play an important role for balance control in visually deprived conditions (King et al., 2019). In summary, all visual deprivation conditions induced instability compared to EO.

### Older versus younger adults

Except the fact that muscle activity of TA showed higher activations in older participants compared to the young group, comparing young versus older subjects did not reveal differences for COP and EMG GM activity in all conditions. This was an unexpected outcome, which does not agree with our hypothesis. These findings contrast previous studies demonstrating poor balance abilities in older compared to young subjects, especially with deprived vision (Toledo & Barela, 2014; Era & Heikkinen, 1985; Connell et al., 2017; Wade et al., 1995). However, there are also comparable studies showing no differences in balance parameters between young and older adults (Hytönen et al., 2009; Prioli, Freitas & Barela, 2005; Whipple et al., 1993; Sundermier et al., 1996), which is in line with our findings.

We particularly expected more instability in our older group due to the normal aging process including age-induced structural and functional changes, such as a reduction of the number of synapses and decreases in signal transmission velocity (Pannese, 2011; Lu et al., 2012). Although the age-induced degenerative processes are related to balance control systems (such as proprioceptors and neurons Shaffer & Harrison, 2007; Pannese, 2011), balance responses depend on the complexity of the balance task (Prioli et al., 2006). It seems that the literature is quite divergent in this context, because the results depend on many other factors, like the complexity level of the balance task (Prioli et al., 2006), age, physical activity level, and history of falls. The bipedal quasi-static balance tasks performed are considered to exhibit low postural demands, even without vision. It was confirmed that the differences in sensory–motor combination of young vs. older adults were observed in more demanding tasks (e.g., dynamic tasks), but not in normal support (e.g., double limb stance on parallel base of support) (Prioli et al., 2006). Therefore, our quasi-static balance results strengthen the notion of a similarly successful central integration of the sensory systems in both age groups. Since our dynamic balance tasks were more demanding than the quasi-static tasks, differences between young and older participants appear more likely. Indeed, Hurley, Rees & Newham (1998) found that demanding tasks were associated with age-related postural differences (although the subjects were active, but there were no further information in terms of the type or frequency of (sportive) activities). However, we did not find such differences regarding dynamic balance. A possible explanation for this absence is that more than 65% of our older participants were physically active, which may account for these results, since physical activity may improve sensory integration for balance control (Prioli, Freitas & Barela, 2005). However, it is important to note that participants’ age may also play a role. Another study found that postural instability was present only in older adults aged 76–90, especially when visual deprivation was imposed (Hytönen et al., 2009). Furthermore, Proske and Gandevia (Proske & Gandevia, 2012) argued that vision plays a major role during standing for adults up to the age of 65 years, but as visual acuity declines, it becomes less important. The mean age of the older participants (sub-groups) from some studies (Hytönen et al., 2009; Berg et al., 1997; Era & Heikkinen, 1985; Hurley, Rees & Newham, 1998) is higher than in our study. Other studies exhibited quite a similar group age structure of their participants (Toledo & Barela, 2014; Hytönen et al., 2009; Wade et al., 1995), and some studies examined participants or sub-groups with a lower mean age than our older adults (Hytönen et al., 2009; Prioli, Freitas & Barela, 2005; Prioli et al., 2006; Era & Heikkinen, 1985; Hurley, Rees & Newham, 1998). Unfortunately, almost all of the above-mentioned studies do not specify whether or not their participants were physically active and if so, what type/s, intensities, or frequencies of activities they were doing. This makes it hard to find similarities or differences with respect to outcomes of other studies. However, it seems most likely that physical activities including their positive effects play a fundamental role, as already mentioned and confirmed (Prioli, Freitas & Barela, 2005).

It is important to mention that a larger TA muscle activation was shown for our older participants, which suggests an additional neuromuscular demand to compensate balance demands. However, this was not reflected in COP responses. Nonetheless, also our older subjects successfully completed all quasi-static and dynamic balance tasks with no falls or invalid trials.

It should also be acknowledged that our study has limitations. We did not measure intrinsic muscles or other small muscles like the peroneus muscles. However, especially when measuring small muscles, it is hard to accurately measure their activity with surface EMG electrodes. In such or similar cases it is usually recommended to use minature electrodes (Saitou et al., 2000) or intra-muscular electrodes (Kelly et al., 2012). Also, there are many possibilities to analyze balance data. It is of course possible that we did not choose the most appropriate parameters in order to assess postural strategies. However, we also analyzed several other COP parameters (e.g., AP and ML range, average velocity, maximum velocity, AP and ML velocity, ellipse area), which support the findings already presented here. Further studies should investigate which modality of physical activity could be the most beneficial for controlling balance at all ages. Furthermore, we suggest carrying out studies to investigate whether there is a “critical age” related to balance control issues, and what interventions are most effective in counteracting this.

## Conclusion

We consistently found larger instability for the visually deprived conditions compared to eyes open, which happened regardless of age. Therefore, we can conclude that the visual system plays an important role for balance control in both age groups. More importantly, the different approaches for visual deprivation did not influence balance outcomes, meaning that results from different deprivation protocols are comparable. Ultimately, COP responses did not differ between young adults and older adults, independent of the level of balance demand (quasi-static or dynamic). Although older participants showed additional neuromuscular demand compared to younger adults for some of the TA muscle activity, we can summarize that there are no major age differences. As our older subjects were physically active, it could be that sportive activities positively influence postural ability. Further studies should investigate whether there is a critical illumination level necessary to prevent postural instabilities, and what interventions or training programs might be most effective in this regard.

##  Supplemental Information

10.7717/peerj.11221/supp-1 Supplemental Information 1 All raw data Group 1 = Young subjects, Group 2 = Older subjects, 0 = Interval 0, 1 = Interval 1, 2 = Interval 2, EO = Eyes open, EC = Eyes closed, BG = Blackout glasses, DR = Dark room.

10.7717/peerj.11221/supp-2 Supplemental Information 2 Questionnaire: English version

10.7717/peerj.11221/supp-3 Supplemental Information 3 Questionnaire: German original

## Additional Information and Declarations

### Data Availability

The following information was supplied regarding data availability: All raw data, including the data used for tables and figures, is available in the Supplementary Files.
