# Supplementary material for: Different visual manipulations have similar effects on quasi-static and dynamic balance responses of young and older people
Source: PeerJ. 2021 May 11;9:e11221. doi: 10.7717/peerj.11221 (PMC8121054; doi:10.7717/peerj.11221)
Supplement: Supplemental Information 2 [file peerj-09-11221-s002.pdf]

Subject Protocol / Questionnaire

Subject ID: [ ]

Date:

Time:

Age:

Test Coordinator:

Height:

Weight:

Shoe Size:

Gender:

Restricted vision (right, left, both): (dpt/96:)

Dominant leg when standing:

1- Did you perform any sportive activity during the last 4 hours? Yes / No

2- Do you suffer from injuries of your lower extremities? Yes / No
If yes, please specify:

3- Do you perform a sportive activity on a regular basis? Yes / No
If yes:
Kind of sportive activity 1: Frequency per week:
Kind of sportive activity 2: Frequency per week:
...

4- Are you taking any medication, which may influence your balance abilities? Yes / No
If yes, please specify:

5- Do you suffer from claustrophobia? Yes / No

6- Additional notes:
