# Supplementary material for: Different visual manipulations have similar effects on quasi-static and dynamic balance responses of young and older people
Source: PeerJ. 2021 May 11;9:e11221. doi: 10.7717/peerj.11221 (PMC8121054; doi:10.7717/peerj.11221)
Supplement: Supplemental Information 3 [file peerj-09-11221-s003.pdf]

Probandenprotokoll / Fragebogen

Probandenkürzel: [ ] Datum:

Uhrzeit:

Alter:

Testleiter:

Körpergröße:

Körpergewicht:

Schuhgröße:

Geschlecht:

Seheinschränkung (rechts, links, beidseitig): (dpt/96:

Standbein:

1- Haben Sie in den letzten 4 Stunden Sport getrieben? Ja / Nein

2- Haben Sie Verletzungen der unteren Extremitäten? Ja / Nein
Wenn ja, welche:

3- Treiben Sie regelmäßig Sport? Ja / Nein
Wenn ja, welchen Sport:
Sportart 1: Häufigkeit pro Woche:
Sportart 2: Häufigkeit pro Woche:
...

4- Nehmen Sie Medikamente ein, die das Gleichgewicht beeinflussen? Yes / No
Wenn ja, welche:

5- Leiden Sie unter Klaustrophobie? Yes / No

6- Zusätzliche Anmerkungen:
